# Epinephrine in pediatric cardiorespiratory arrest: when and how much?

**DOI:** 10.31744/einstein_journal/2020RW5055

**Published:** 2020-01-23

**Authors:** João Carlos Pina Faria, Camila Augusta Victorino, Monica Akemi Sato

**Affiliations:** 1 Faculdade de Medicina do ABC Centro Universitário Saúde ABC Santo AndréSP Brazil Faculdade de Medicina do ABC, Centro Universitário Saúde ABC, Santo André, SP, Brazil.; 2 Universidade Nove de Julho São PauloSP Brazil Universidade Nove de Julho, São Paulo, SP, Brazil.

**Keywords:** Heart arrest, Out-of-hospital cardiac arrest, Child, Adolescent, Epinephrine/administration & dosage, Survival

## Abstract

The objective of the present study was to assess the efficacy of different doses, times for infusion of the first dose, intervals of administration of subsequent doses, and number of epinephrine doses in the survival of children and adolescents who went into cardiorespiratory arrest. It is a review study with data from the PubMed^Ⓡ^/MEDLINE^Ⓡ^database. The search was for articles published from January 1^st^, 2000 to February 10, 2019, with a sample of patients aged under 18 years, published in English, Portuguese and Spanish. We found 222 articles, of which 16 met the inclusion criteria of the study. The first dose should be given as soon as possible. The standard dose (0.01mg/kg) has a better outcome when compared to the higher dose (0.1mg/kg). There is an iⓇverse relation between the number of epinephrine doses and survival. The interval currently recommended between doses has lower survival when compared to larger intervals. The dosage recommended by the American Heart Association presents a better outcome for survival, but the interval between doses and the maximum number of doses should be better assessed.

## INTRODUCTION

Epinephrine was isolated in 1900.^1^It began to be used for treating cardiorespiratory arrest in the 1960´s.^2^The American Heart Association (AHA) currently indicates epinephrine to be administered as soon as possible at a dose of 0.01mg/kg (maximum of 1mg), and at 3-to-5-minute intervals between subsequent doses.^3^The alpha-adrenergic effect, with increase in aortic diastolic pressure and in coronary blood flow, prevails upon standard dose.^4^

Delay in the administration of epinephrine is associated with decreased survival.^5^There is a direct relation between time to administration of epinephrine during cardiopulmonary resuscitation for survival in children.^6 , 7^ When compared to placebo, moderate quality evidence has shown that the standard dose of epinephrine improved return of spontaneous circulation, and survival up to hospital discharge for individuals who had an out-of-hospital cardiac arrest.^8^

During Advanced Life Support maneuvers using epinephrine, the perfusion pressure of the brain and of other organs remains low, until the return of spontaneous circulation.^9^ Studies in animals indicate that epinephrine can reduce microcirculation blood flow, which can cause organ damage.^10^

Considering adults who received epinephrine stratified by rhythms, those non-shockable presented increased survival.^11^ These rhythms are more frequent in pediatrics.^12^

Most studies involving epinephrine in cardiorespiratory arrest were performed in adults. American Heart Association recommendations in pediatrics are similar to those for adults, except for dosage of epinephrine, and indication in cardiorespiratory arrest and the same administration time interval remain. It is, however, important to determine if the protocol that is being followed in pediatrics has consequences that may or may not be the most appropriate for patients.

## OBJECTIVE

To assess the efficacy of different doses, times for infusion of the first dose, intervals of administration of subsequent doses, and the number of epinephrine doses in the survival of children and adolescents who had an in-hospital or out-of-hospital cardiorespiratory arrest.

## METHODS

A systematic literature review was carried out for articles on the PubMed+/MEDLINE^Ⓡ^ database. The keywords “cardiac arrest”, “children” and “epinephrine”, combined with the “AND” boolean operator were used.

Following, four PubMed^Ⓡ^ filters were considered: period (Publication date from 2000/01/01 to 2019/02/10), studies in Humans, articles in English, Portuguese or Spanish and age group (child: birth-18 years). Titles and summary of the articles selected were assessed. Studies that assessed the efficacy of epinephrine to treat cardiorespiratory arrest in the pediatric age group were included.

## RESULTS

Of the 222 articles found, 126 were excluded upon initial analysis using the four PubMed^Ⓡ^ filters: 89 studies published before January 1, 2000; 14 studies performed with animals; 14 studies with languages other than English, Spanish or Portuguese; 9 studies performed with patients over 18 years of age.

**Figure 1:**
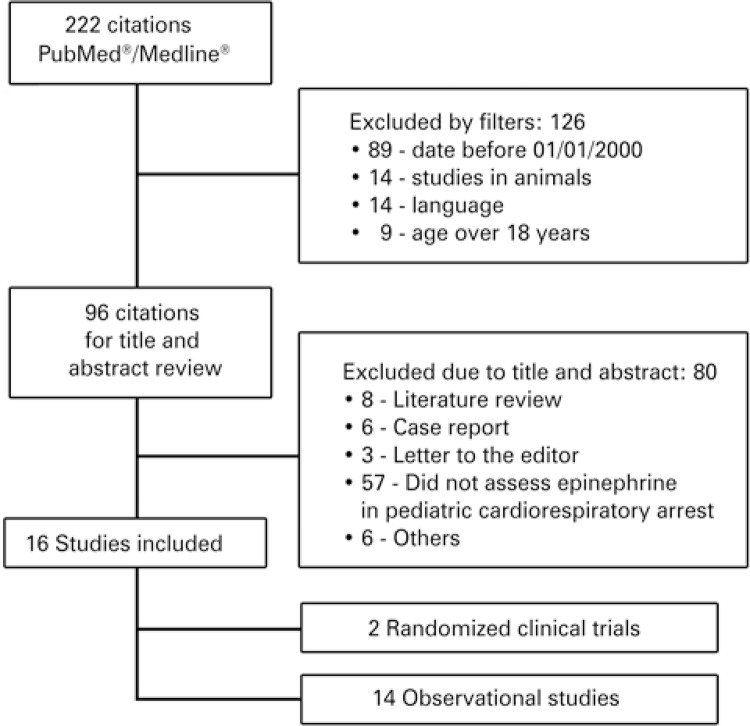
Study flow chart

The remaining 96 articles were assessed. Of these, 95 were in English and one in Spanish. Eighty articles were excluded because they did not describe the efficacy of epinephrine to treat cardiorespiratory arrest in children, regarding survival ( Figure 1 ).

In the second stage, 8 literature reviews, 6 case reports, 3 letters to the editor were excluded, as were 57 studies that did not assess specifically epinephrine in pediatric cardiorespiratory arrest, and 6 were excluded for other reasons.

There were 16 studies^5 , 13 - 27^ selected among those found on the PubMed^Ⓡ^/MEDLINE^Ⓡ^ database ( Table 1 ). Although there was no search for articles on other databases, all of these articles were cited on the Scopus database, and 15 were cited on the ISI Web of Science database. Only the *Anales de Pediatría* ^27^ journal was not present in the ISI Web of Science database. Two studies were randomized clinical trials, and 14 were observational studies.

**Table 1:** Studies selected for review

Reference	Type of study	Type of cardiorespiratory arrest	Number of events	Objective	Conclusion
Fukuda et al.^(5)^	Observational	Out-of-hospital	225	Assess time to first epinephrine dose and survival	The shorter the time to first epinephrine dose, the higher the survival
Andersen et al.^(13)^	Observational	In-hospital	1,558	Assess time to first epinephrine dose with survival and neurological prognosis	The shorter the time to first epinephrine dose, the higher the survival, and the better the neurological prognosis
Lin et al.^(14)^	Observational	Out-of-hospital trauma	388	Assess time to first epinephrine dose with survival and neurological prognosis	Early treatment with epinephrine may not provide benefits in cases of trauma
Hoyme et al.^(15)^	Observational	In-hospital	1,630	Assess different intervals between doses of epinephrine and survival	Administration intervals longer than those currently recommended present higher survival
Meert et al.^(16)^	Observational	Out-of-hospital	295	Assess management during cardiorespiratory arrest with survival and neurological prognosis	The higher the number of doses of epinephrine, the lower the survival, and the worse the neurological prognosis
Moler et al.^(17)^	Observational	Out-of-hospital	138	Assess different managements to treat cardiorespiratory arrest and survival	The higher the number of doses of epinephrine, the lower the survival
de Mos et al.^(18)^	Observational	In-hospital	91	Assess different managements to treat cardiorespiratory arrest and survival	The higher the number of doses of epinephrine, the lower the survival
Young et al.^(19)^	Observational	Out-of-hospital	601	Describe epidemiological characteristics, survival rates and neurological outcomes	The higher the number of doses of epinephrine, the lower the survival, and the worse the neurological prognosis
Checchia et al.^(20)^	Observational	Out-of-hospital	24	Determine if the measurement of heart troponin I in children in cardiorespiratory arrest outside the hospital anticipates the severity of the myocardial lesion	The higher the number of doses of epinephrine, the lower the survival
Donoghue et al.^(21)^	Observational	In-hospital not trauma	16,834 (16,245 adults and 537 children)	Compare results of cardiopulmonary resuscitation for pediatric patients and adults, and identify factors associated with differences in results between children and adults	More epinephrine was used in children In the multivariate analysis, age did not present association regarding survival
Enright et al.^(22)^	Observational	Out-of-hospital during physical exercise	9	Determine if long-term survival is influenced by specific resuscitation interventions	Children who went into cardiorespiratory arrest during physical exercise present mainly non-shockable rhythms, and epinephrine, in this scenario, may be harmful
Tibballs et al.^(23)^	Observational	In-hospital	111	Assess the impact of different factors in prognosis of cardiorespiratory arrest in children	Doses of epinephrine above 0.015mg/kg present lower survival
Perondi et al.^(24)^	Randomized	In-hospital	68	Compare efficacy between standard dose and high dose of epinephrine in hospital pediatric cardiorespiratory arrest	Data suggest that treatment with high doses may be worse than treatment with standard dose
Guay et al.^(25)^	Observational	In-hospital	203	Assess efficacy of advanced life support interventions in pediatric cardiorespiratory arrest	For epinephrine administered by the intravenous route, the standard dose of 0.01mg/kg seems appropriate as an initial dose
Patterson et al.^(26)^	Randomized	Out-of-hospital	230	Assess if a high dose of epinephrine used during out-of-hospital cardiorespiratory arrest refractory to pre-hospital interventions improves return of spontaneous circulation, survival and neurological prognosis	A high-dose of epinephrine does not improve or decrease return of spontaneous circulation, survival and neurological prognosis in comparison with standard dose in out-of-hospital cardiorespiratory arrest
Rodríguez Núñez et al.^(27)^	Observational	Intra and out-of-hospital	92	Assess the impact on survival of epinephrine (intravenous or intraosseous) in high-dose in comparison to standard dose in children in cardiorespiratory arrest	There was no difference in the two groups regarding return of spontaneous circulation, total resuscitation time, neurological status at the end of the episode and survival to hospital discharge and in 1-year follow-up

## DISCUSSION

The relevant aspects for the comparison of articles found in the literature using the chosen keywords are the following:

### Time to administration of first epinephrine dose

Three studies assessed if time to administration of first epinephrine dose influenced survival.

Fukuda et al., analyzed 225 children between 1 and 17 years of age who had an out-of-hospital cardiorespiratory arrest in Japan, using retrospective data. There was a direct relation between shorter time to administration of epinephrine and higher 30-day survival (p<0.0001).^5^

Andersen et al., assessed 1,558 children aged under 18 years, who had an in-hospital cardiorespiratory arrest in the United States, in a retrospective cohort. Similar to the previous study, there was also a relation between shorter time to administration and higher survival (p<0.001).^13^ The authors also reported that the neurological prognosis was better in the group with shorter time to administration.^13^However, Lin et al., observed that time to the first dose of epinephrine did not influence survival or neurological prognosis, when they studied 388 children aged under 18 years, seen at 3 emergency centers of Taiwan (p=0.234).^14^

The different studies that assessed time to administration of first epinephrine dose were carried out with an expressive number of children, albeit ages varied from infants to 18 years. In the study of Fukuda et al.,^5^ there was a predominance of adolescents over 12 years (76.9%). The study of Andersen et al.,^13^ comprised a sample of children with a median age of 9 months. Lin et al.,^14^ included 63.4% of children and adolescents aged over 10 years. Despite the wide range of age groups in the different studies, the factor did not seem to be a determinant of the differences observed in the study by Lin et al.,^14^ in comparison to those of Fukuda et al.,^5^ and Andersen et al.^13^ Therefore, the rationale for this conflicting result is the fact that the study of Lin et al.,^14^ only assessed out-of-hospital cardiorespiratory arrest secondary to trauma, while the other two studies assessed all causes of cardiorespiratory arrest.

A study performed with 35,065 adults who went into a non-trauma out-of-hospital cardiorespiratory arrest showed that when Advanced Life Support (epinephrine, airway establishment and manual defibrillation) began early, there was higher survival of victims, when compared to Basic Life Support (cardiopulmonary resuscitation and automatic external defibrillator).^28^

### Time interval between doses of epinephrine

A retrospective review of the AHA Get With The Guidelines^Ⓡ^-Resuscitation registry, with 1,630 children aged under 18 years, who had an in-hospital cardiorespiratory arrest, showed that intervals between epinephrine doses longer (>5 to <8 minutes, and 8 to <10 minutes) than those currently recommended by the AHA attained better results in survival to hospital discharge.^15^A study performed in 2014 in an adult population found similar results after assessing 20,909 in-hospital cardiorespiratory arrest.^29^

Although an important finding with a high number sample, it is the only study up to present in the literature showing findings different from those recommended.

### Number of doses of epinephrine administered

Seven studies found an inverse relation between number of doses of epinephrine and survival.

Data of the Therapeutic Hypothermia after Pediatric Cardiac Arrest Out-of-Hospital Trial on 36 pediatric intensive care units (ICU) in Canada and the United States, with 295 children over 48 hours of life and under 18 years of age who had an out-of-hospital cardiorespiratory arrest were submitted to secondary analysis. The study showed 80% survival when epinephrine did not have to be administered, 54% with one dose, 41% with two doses, 38% with three doses, 36% with four doses, 11% with five or more doses.^16^ This study found the same relation between the number of doses and the neurological prognosis defined by the second edition of the Vineland Adaptive Behavior Scales (VABS-II).^16^

A retrospective cohort, with 138 children with more than 24 hours of life and under 18 years, at 15 hospitals, victims of out-of-hospital cardiorespiratory arrest, showed that the number of doses of epinephrine was inversely associated with hospital discharge (p<0.01).^17^ Only seven of the 46 patients who received more than three doses of epinephrine survived.^17^ The maximum number of doses of adrenalin received by a normal survivor was five.^17^

A retrospective study with 91 children under 18 years who had a cardiorespiratory arrest in the pediatric ICU showed that the survival of children who received from two to three doses was 48%, and 13% for those who received four or more doses.^18^

The secondary analysis of data of a prospective study carried out from 1994 to 1997 assessed 601 out-of-hospital cardiorespiratory arrest in children under 12 years, in two cities in California.^19^ Children who received more than three doses of epinephrine or more than 31 minutes of cardiopulmonary resuscitation did not survive.^19^

A prospective observational study on 24 children, aged between 8 months and 17 years, admitted to the pediatric ICU and who had an out-of-hospital cardiorespiratory arrest, concluded that survivors received 1.3 +/- 2.2 doses of epinephrine in comparison to 2.9 +/- 1.6 doses received by non-survivors (p=0.02).^20^

A retrospective 10-year cohort assessed 16,834 non-trauma cardiorespiratory arrest (537 in children) seen at the Emergency Room.^21^ The median dose of epinephrine was 3 (zero to 15) in children, and 2 (zero to 9) in adults (p<0.001).^21^Adults presented more frequent return of spontaneous circulation (53% *versus* 47%; p=0.02), 24-hour survival (35% *versus* 30%; p=0.02) and survival to discharge (23% *versus* 20%; p=NS); however, in the multivariate analysis, age did not associate with outcomes.^21^

An assumption to explain lower survival with increase in the number of doses of epinephrine is that, the longer cardiorespiratory arrest time, the more doses are administered. Thus, lower survival may be related to duration of cardiorespiratory arrest and not necessarily to an adverse effect of the drug. Another factor that may also contribute to survival of children victims of cardiorespiratory arrest is heart rhythm. Children who initially presented a shockable rhythm cardiorespiratory arrest had higher survival.^30^ A prospective observational study carried out in Sidney observed nine children under 16 years of age, who had an out-of-hospital cardiorespiratory arrest during physical activity.^22^The six survivors had a shockable rhythm cardiorespiratory arrest and five of them did not receive epinephrine. The three that died had non-shockable rhythm cardiorespiratory arrest.^22^ Despite the findings of this study, the number of children assessed was very limited, which did not render an appropriate statistical analysis to reach conclusions that may be reflected population wise.

### Dosage of epinephrine administered

Five studies that assessed the relation between the dose of epinephrine administered and survival were found. Three concluded that the standard dose (0.01mg/kg) attains better results. Two concluded that the standard dose or doses above standard have the same survival outcome. No study has shown improvement in survival with super dosages of epinephrine.

A prospective study with 111 children concluded that doses above 0.015mg/kg administered for non-shockable rhythms may lead to secondary ventricular fibrillation, which has a worse outcome than primary ventricular fibrillation.^23^

A prospective, randomized, double-blind study compared high-doses of epinephrine (0.1mg/kg) with the standard dose of epinephrine as salvage therapy for hospital cardiorespiratory arrest in children after failure of an initial standard dose (n=68).^24^ The two treatment groups did not differ significantly in terms of return of spontaneous circulation rate (which occurred in 20 patients in the high-dose group, and in 21 in the standard dose group).^24^ However, the 24-hour survival rate was lower in the high dose epinephrine group as salvage therapy in comparison to the standard dose group.^24^One of the 34 patients of the high-dose group survived 24 hours, in contrast to 7 of the 34 standard dose patient group (p=0.05).^24^

A retrospective study compared standard dose, low doses and high doses of epinephrine in pediatric cardiorespiratory arrest. There was no immediate survival or after 24 hours in the group that received a dose below 0.0018mg/kg.^25^In the group that received doses above standard, there was no immediate survival in children who received more than 0.0357mg/kg, and there were no survivors in 24 hours among children who received a dose above 0.019mg/kg.^25^

A multicenter randomized controlled study conducted in seven hospitals with 230 patients under 22 years compared the standard dose (n=86) with a dose ten-fold higher of epinephrine (n=127).^26^There was no statistically significant difference between both groups regarding outcome (return of spontaneous circulation, 24-hour survival, survival to discharge and neurological prognosis).^26^

A multicenter prospective study analyzed 92 children between 7 days of life and 17 years, victims of cardiorespiratory arrest, in two groups.^27^ The first group (n=12) received standard doses of epinephrine while the second (n=80) received the standard dose first, and the remaining doses ten-fold higher.^27^ There was no difference between the two groups regarding return of spontaneous circulation, total resuscitation time, neurological status at the end of the episode, and survival to hospital discharge and in the one-year follow-up.^27^ The studies assessed in this review showed no advantages in changing the standard dose recommended by the AHA.

## CONCLUSION

There are few articles published assessing the use of epinephrine in pediatrics. After assessing the studies of this review, we have concluded that epinephrine should be administered in cardiorespiratory arrest in children as soon as possible. The dose should be standard (0.01mg/kg). Despite studies having showed lower survival with increase in number of epinephrine doses, the fact can be explained by other reasons, such as cardiopulmonary resuscitation time and the initial rhythm of the cardiorespiratory arrest. Only one study assessed the interval between doses of epinephrine and concluded that the interval currently recommended (3 to 5 minutes) leads to lower survival when compared to longer intervals, as it has already been demonstrated for adults, suggesting that less epinephrine (cumulative dose or frequency) can be beneficial in in-hospital pediatric cardiorespiratory arrest.

More studies, preferably randomized clinical trials, need to be performed to better understand how to adjust intervals and maximum number of doses of epinephrine.
