# Supplementary material for: Epinephrine in pediatric cardiorespiratory arrest: when and how much?
Source: Einstein (Sao Paulo). 2020 Jan 23;18:eRW5055. doi: 10.31744/einstein_journal/2020RW5055 (PMC6986458; doi:10.31744/einstein_journal/2020RW5055)
Supplement: Translation

# Translation: Epinefrina na parada cardiorrespiratória pediátrica: quando e quanto?

**Authors:** João Carlos Pina Faria, Camila Augusta Victorino, Monica Akemi Sato

**Affiliations:** 1 Brasil Faculdade de Medicina do ABC, Centro Universitário Saúde ABC, Santo André, SP, Brasil.; 2 Brasil Universidade Nove de Julho, São Paulo, SP, Brasil.

**Keywords:** Parada cardíaca, Parada cardíaca extra-hospitalar, Criança, Adolescente, Epinefrina/administração & dosagem, Sobrevida

## Abstract

O objetivo deste estudo foi avaliar a eficácia de diferentes doses, tempos para infusão da primeira dose, intervalos de administração de doses subsequentes e número de doses de epinefrina na sobrevida de crianças e adolescentes que sofreram parada cardiorrespiratória. Trata-se de estudo de revisão, cujas buscas foram feitas na base de dados PubMed^Ⓡ^/MEDLINE^Ⓡ^. Foram selecionados artigos publicados de 1° de janeiro de 2000 até 10 de fevereiro de 2019, realizados em menores de 18 anos de idade, publicados em inglês, português e espanhol. Foram encontrados 222 artigos, dos quais 16 atenderam os critérios de inclusão no estudo. A primeira dose deve ser aplicada o mais rápido possível. A dose padrão (0,01mg/kg) apresenta melhor desfecho quando comparada à dose alta (0,1mg/kg). Houve relação inversa entre número de doses de epinefrina e sobrevida. O intervalo entre doses recomendado atualmente apresenta menor sobrevida quando comparado a intervalos maiores. A dose recomendada pela *American Heart Association* apresenta melhor desfecho para sobrevida, porém o intervalo entre doses e o número máximo de doses devem ser melhor avaliados.

## INTRODUÇÃO

A epinefrina foi isolada em 1900.^1^Na década de 1960, passou a ser utilizada para o tratamento da parada cardiorrespiratória.^2^Atualmente, a *American Heart Association* (AHA) indica que a epinefrina deve ser administrada o mais rápido possível, na dosagem de 0,01mg/kg (máximo 1mg), e com intervalos de 3 a 5 minutos entre as doses subsequentes.^3^Na dose padrão, predomina o efeito alfa-adrenérgico, com aumento da pressão diastólica aórtica e do fluxo sanguíneo coronariano.^4^

O atraso na administração de epinefrina está associado com diminuição da sobrevida.^5^Há relação direta entre o tempo para a administração da epinefrina durante a ressuscitação cardiopulmonar com a sobrevida da criança.^6 , 7^Comparado ao placebo, evidências de qualidade moderada mostraram que a epinefrina, na dose padrão, melhorou o retorno da circulação espontânea, e a sobrevivência até a alta hospitalar em pessoas com parada cardíaca extra-hospitalar.^8^

Durante as manobras do Suporte Avançado de Vida com o uso de epinefrina, a pressão de perfusão cerebral e de outros órgãos permanece baixa, até o retorno da circulação espontânea.^9^Estudos com animais indicam que a epinefrina pode reduzir o fluxo sanguíneo na microcirculação, o que pode causar lesões nos órgãos.^10^

Em adultos que receberam epinefrina estratificados por ritmos, os não desfibriláveis tiveram aumento na sobrevida.^11^Estes ritmos são os mais frequentes em pediatria.^12^

A maior parte das pesquisas envolvendo epinefrina na parada cardiorrespiratória foi realizada em adultos. As recomendações da AHA em pediatria são semelhantes às de adultos, exceto pela dosagem de epinefrina, mantendo-se a indicação em parada cardiorrespiratória e o mesmo intervalo de tempo de administração. Porém, torna-se importante determinar se o protocolo que vem sendo seguido em pediatria apresenta repercussões que poderiam ou não ser as mais adequadas ao paciente.

## OBJETIVO

Avaliar a eficácia de diferentes doses, tempos para infusão da primeira dose, intervalos de administração de doses subsequentes, e número de doses de epinefrina na sobrevida de crianças e adolescentes que sofreram parada cardiorrespiratória extra e intra-hospitalar.

## MÉTODOS

Realizou-se revisão sistemática de literatura, por meio de artigos da base de dados PubMed^Ⓡ^/MEDLINE^Ⓡ^. Utilizaram-se os descritores “ *cardiac arrest* ”, “ *children* ” e “ *epinephrine* ”, combinados com o operador booleano “ *AND* ”.

Em seguida, utilizaram-se quatro filtros do PubMed^Ⓡ^: período (Data de publicação: 01/01/2000 até 10/02/2019), estudos em humanos ( *Humans* ), artigos nos idiomas inglês, português e espanhol ( *English, Portuguese, Spanish* ) e faixa etária de zero a 18 anos ( *Child: birth-18 years* ). Os artigos selecionados tiveram título e resumo avaliados. Foram incluídos os estudos que avaliaram a eficácia da epinefrina no tratamento da parada cardiorrespiratória pediátrica.

## RESULTADOS

Foram encontrados 222 artigos e, destes, 126 foram excluídos na análise inicial devido aos quatro filtros do PubMed^Ⓡ^: 89 estudos publicados antes de 1° de janeiro de 2000; 14 estudos realizados em animais; 14 estudos com idiomas diferentes do inglês, espanhol e português; 9 estudos realizados em pacientes com idade acima de 18 anos.

Os 96 artigos restantes foram avaliados. Destes, 95 estavam no idioma inglês e um em espanhol. Foram excluídos 80 artigos, pois não descreveram a eficácia da epinefrina no tratamento da parada cardiorrespiratória pediátrica em relação à sobrevida ( Figura 1 ).

**Figura 1:** Fluxograma do estudo

Na segunda etapa, foram excluídos 8 revisões de literatura, 6 relatos de caso, 3 cartas ao editor, 57 estudos que não avaliaram especificamente epinefrina na parada cardiorrespiratória pediátrica e 6 por outros motivos.

Foram selecionados 16 estudos^5 , 13 - 27^encontrados na base de dados PubMed^Ⓡ^/MEDLINE^Ⓡ^( Tabela 1 ). Embora não tenha sido realizada busca de artigos em outras bases de dados, todos estavam citados na base de dados Scopus e 15 estavam na base ISI *Web of Science* . Apenas o periódico *Anales de Pediatría* ^27^não estava presente na base de dados ISI *Web of Science* . Dois estudos eram ensaios clínicos aleatorizados e 14 eram observacionais.

**Tabela 1:** Estudos selecionados para a revisão

Referência	Tipo de estudo	Tipo de parada cardiorrespiratória	Número de eventos	Objetivo	Conclusão
Fukuda et al.^(5)^	Observacional	Extra-hospitalar	225	Avaliar tempo para primeira epinefrina e sobrevida	Quanto menor o tempo para a primeira epinefrina, maior a sobrevida
Andersen et al.^(13)^	Observacional	Intra-hospitalar	1.558	Avaliar tempo para primeira epinefrina com sobrevida e prognóstico neurológico	Quanto menor o tempo para a primeira epinefrina, maior a sobrevida e melhor o prognóstico neurológico
Lin et al.^(14)^	Observacional	Traumática extra-hospitalar	388	Avaliar tempo para primeira epinefrina com sobrevida e prognóstico neurológico	O tratamento precoce com epinefrina pode não proporcionar benefícios em casos traumáticos
Hoyme et al.^(15)^	Observacional	Intra-hospitalar	1.630	Avaliar diferentes intervalos entre doses de epinefrina e sobrevida	Intervalos de administração maiores que os recomendados atualmente apresentam maior sobrevida
Meert et al.^(16)^	Observacional	Extra-hospitalar	295	Avaliar a conduta durante a parada cardiorrespiratória com sobrevida e prognóstico neurológico	Quanto maior o número de doses de epinefrina, menor a sobrevida e pior o prognóstico neurológico
Moler et al.^(17)^	Observacional	Extra-hospitalar	138	Avaliar diferentes condutas no tratamento da parada cardiorrespiratória e sobrevida	Quanto maior o número de doses de epinefrina, menor a sobrevida
de Mos et al.^(18)^	Observacional	Intra-hospitalar	91	Avaliar diferentes condutas no tratamento da parada cardiorrespiratória e sobrevida	Quanto maior o número de doses de epinefrina, menor a sobrevida
Young et al.^(19)^	Observacional	Extra-hospitalar	601	Descrever as características epidemiológicas, as taxas de sobrevivência e os desfechos neurológicos	Quanto maior o número de doses de epinefrina, menor a sobrevida e pior o prognóstico neurológico
Checchia et al.^(20)^	Observacional	Extra-hospitalar	24	Determinar se a medida da troponina I cardíaca em crianças com parada cardiorrespiratória fora do hospital prevê a gravidade da lesão miocárdica	Quanto maior o número de doses de epinefrina, menor a sobrevida
Donoghue et al.^(21)^	Observacional	Não traumática intra-hospitalar	16.834 (16.245 adultos e 537 crianças)	Comparar os resultados da ressuscitação cardiopulmonar para pacientes pediátricos e adultos e identificar fatores associados às diferenças nos resultados entre crianças e adultos	Utilizou-se mais epinefrina em crianças Na análise multivariada, a idade não teve associação na sobrevida
Enright et al.^(22)^	Observacional	Extra-hospitalar durante exercício físico	9	Determinar se a sobrevivência a longo prazo é influenciada por intervenções específicas de ressuscitação	Crianças que sofrem parada cardiorrespiratória durante exercício físico apresentam principalmente ritmos desfibriláveis e a epinefrina, nessa situação, pode ser maléfica
Tibballs et al.^(23)^	Observacional	Intra-hospitalar	111	Avaliar o impacto de diferentes fatores no prognóstico da parada cardiorrespiratória em crianças	Doses de epinefrina superiores a 0,015mg/kg apresentam menor sobrevida
Perondi et al.^(24)^	Aleatorizado	Intra-hospitalar	68	Comparar a eficácia entre a dose padrão e dose alta de epinefrina em parada cardiorrespiratória pediátrica hospitalar	Os dados sugerem que a terapia com doses elevadas pode ser pior do que a terapia de dose padrão
Guay et al.^(25)^	Observacional	Intra-hospitalar	203	Avaliar a eficácia das intervenções avançadas de suporte de vida na parada cardiorrespiratória pediátrica	Para a epinefrina administrada por via intravenosa, a dose padrão de 0,01mg/kg parece apropriada como a primeira dose
Patterson et al.^(26)^	Aleatorizado	Extra-hospitalar	230	Avaliar se a dose elevada de epinefrina utilizada durante a parada cardiorrespiratória extra-hospitalar refratária às intervenções pré-hospitalares melhora o retorno da circulação espontânea, a sobrevida e o prognóstico neurológico	A dose alta de epinefrina não melhora e nem diminui o retorno da circulação espontânea, a sobrevida e o prognóstico neurológico em comparação com dose padrão na parada cardiorrespiratória extra-hospitalar
Rodríguez Núñez et al.^(27)^	Observacional	Intra e extra-hospitalar	92	Avaliar o impacto na sobrevida de epinefrina (intravenosa ou intraóssea) em dose elevada em comparação com dose padrão em crianças com parada cardiorrespiratória	Não houve diferença nos dois grupos em relação ao retorno da circulação espontânea, tempo de ressuscitação total, estado neurológico no final do episódio e sobrevivência à alta hospitalar e em 1 ano de acompanhamento

## DISCUSSÃO

Considerando-se os artigos encontrados na literatura a partir dos descritores escolhidos, os aspectos relevantes a serem comparados são os seguintes.

### Tempo para administração da primeira epinefrina

Três estudos avaliaram se o tempo para a administração da primeira epinefrina influenciava na sobrevida.

Fukuda et al., analisaram 225 crianças entre 1 e 17 anos que sofreram parada cardiorrespiratória extra-hospitalar no Japão, por meio de levantamento retrospectivo. Houve relação direta entre o menor tempo para administração da epinefrina e maior sobrevida em 30 dias (p<0,0001).^5^

Andersen et al., avaliaram 1.558 crianças menores de 18 anos, que sofreram parada cardiorrespiratória intra-hospitalar nos Estados Unidos, por meio de um coorte retrospectivo. Assim como o estudo anterior, também houve relação entre menor tempo da administração com maior sobrevida (p<0,001).^13^Este estudo também relatou que o prognóstico neurológico foi melhor no grupo com menor tempo de administração.^13^Entretanto, Lin et al., verificaram que o tempo para a primeira dose de epinefrina não influenciou na sobrevida e nem no prognóstico neurológico, ao estudarem 388 crianças menores de 18 anos atendidas em 3 centros de emergências de Taiwan (p=0,234).^14^

Os diferentes estudos que avaliaram o tempo para a administração da primeira epinefrina foram realizados com número expressivo de crianças, porém com idades que podem ter variado desde bebês até 18 anos. O estudo de Fukuda et al.,^5^apresentou predomínio de adolescentes acima de 12 anos (76,9%). O estudo de Andersen et al.,^13^era constituído por uma amostra de crianças com mediana de 9 meses de idade. Lin et al.,^14^apresentaram 63,4% de crianças e adolescentes com idades superiores a 10 anos. Apesar da dispersão da faixa etária nos diferentes estudos, este fator não parece ter sido determinante nas diferenças observadas no estudo de Lin et al.,^14^comparado aos de Fukuda et al.,^5^e Andersen et al.^13^Portanto, o fator que pode justificar este resultado conflitante é o fato de o estudo de Lin et al.,^14^avaliar somente parada cardiorrespiratória extra-hospitalar secundária ao trauma, enquanto os outros dois estudos avaliaram todas as causas de parada cardiorrespiratória.

Estudo realizado com 35.065 adultos que sofreram parada cardiorrespiratória extra-hospitalar não traumática demonstrou que quando o Suporte Avançado de Vida (epinefrina, obtenção de vias aéreas e desfibrilação manual) era aplicado precocemente, havia aumento na sobrevida da vítima, quando comparado ao atendimento somente com Suporte Básico de Vida (ressuscitação cardiopulmonar e desfibrilador externo automático).^28^

### Intervalo de tempo entre as doses de epinefrina

Uma revisão retrospectiva do registro da AHA *Get With The Guidelines* ^Ⓡ^ *-Resuscitation* , com 1.630 crianças menores de 18 anos que sofreram parada cardiorrespiratória intra-hospitalar, evidenciou que intervalos entre as doses de epinefrina maiores (>5 a <8 minutos, e 8 a <10 minutos) que as recomendadas atualmente pela AHA apresentam melhores resultados de sobrevida à alta hospitalar.^15^Estudo realizado em 2014 na população adulta encontrou resultados semelhantes após avaliar 20.909 parada cardiorrespiratória intra-hospitalares.^29^

Embora se trata de um achado importante com número amostral elevado, este é o único estudo na literatura que evidencia achados distintos daqueles preconizados até o momento.

### Número de doses de epinefrina administrada

Sete estudos encontraram relação inversa entre número de doses de epinefrina e sobrevida.

Foi realizada análise secundária de dados do *Therapeutic Hypothermia after Pediatric Cardiac Arrest Out-of-Hospital Trial* , de 36 unidades de terapia intensiva (UTI) pediátricas no Canadá e Estados Unidos, com 295 crianças maiores de 48 horas de vida e menores de 18 anos que sofreram parada cardiorrespiratória extra-hospitalar. Este estudo evidenciou 80% de sobrevida quando a epinefrina não precisou ser administrada, 54% com uma dose, 41% com duas doses, 38% com três doses, 36% com quatro doses, 11% com cinco ou mais doses.^16^Este estudo encontrou a mesma relação entre o número de doses e o prognóstico neurológico definido pela segunda edição da *Vineland Adaptive Behavior Scales* (VABS-II).^16^

Coorte retrospectiva, com 138 crianças maiores de 24 horas de vida e menores de 18 anos, de 15 hospitais, vítimas de parada cardiorrespiratória extra-hospitalar, demonstrou que o número de doses de epinefrina foi inversamente associado à alta hospitalar (p<0,01).^17^Entre os 46 pacientes que receberam mais que três doses de epinefrina, apenas sete sobreviveram.^17^O número máximo de doses de adrenalina recebidas em um sobrevivente normal foi cinco.^17^

Estudo retrospectivo com 91 crianças menores de 18 anos que sofreram parada cardiorrespiratória em UTI pediátrica verificou que a sobrevida das crianças que receberam de duas a três doses foi de 48%, enquanto as que receberam quatro ou mais doses foi de 13%.^18^

Análise secundária de dados de um estudo prospectivo realizado de 1994 a 1997 avaliou 601 parada cardiorrespiratória extra-hospitalares de crianças menores de 12 anos em duas cidades da Califórnia.^19^Crianças que receberam mais de três doses de epinefrina ou mais de 31 minutos de ressuscitação cardiopulmonar não sobreviveram.^19^

Estudo prospectivo observacional com 24 crianças hospitalizadas na UTI pediátrica e que sofreram parada cardiorrespiratória extra-hospitalar, com idades entre 8 meses e 17 anos, concluiu que os sobreviventes receberam 1,3 +/- 2,2 doses de epinefrina em comparação com 2,9 +/- 1,6 doses para não sobreviventes (p=0,02).^20^

Uma coorte retrospectiva com 10 anos de duração avaliou 16.834 parada cardiorrespiratória (537 em crianças) não traumáticas atendidas em departamento de emergência.^21^A mediana das doses de epinefrina foi de 3 (zero a 15) em crianças e 2 (zero a 9) em adultos (p<0,001).^21^Os adultos tiveram retorno mais frequente da circulação espontânea (53% *versus* 47%; p=0,02), sobrevida em 24 horas (35% *versus* 30%; p=0,02) e sobrevida para alta (23% *versus* 20%; p=NS); porém, na análise multivariada, a idade não teve associação com os desfechos.^21^

Uma hipótese que pode explicar a menor sobrevida com o aumento do número de doses de epinefrina é que, quanto maior o tempo de parada cardiorrespiratória, mais doses serão administradas. Desta forma, a menor sobrevida pode estar relacionada ao tempo de duração da parada cardiorrespiratória e não necessariamente a um efeito adverso da droga. Outro fator que também pode contribuir com a sobrevida de crianças vítimas de parada cardiorrespiratória é o ritmo cardíaco. Crianças que apresentam inicialmente ritmo chocável de parada cardiorrespiratória apresentam maior sobrevida.^30^Estudo observacional prospectivo realizado em Sidney observou nove crianças menores de 16 anos que sofreram parada cardiorrespiratória extra-hospitalar durante atividade física.^22^Os seis sobreviventes tiveram parada cardiorrespiratória por ritmo chocável e cinco destes não receberam epinefrina. Os três que faleceram tiveram parada cardiorrespiratória por ritmo não chocável.^22^Apesar dos achados deste estudo, o número de crianças avaliadas é muito limitado, o que inviabiliza a avaliação estatística adequada que permita chegar a conclusões que se reflitam em termos populacionais.

### Dosagem da epinefrina administrada

Foram encontrados cinco estudos que avaliaram a relação entre dose da epinefrina administrada e sobrevida. Três concluíram que a dose padrão (0,01mg/kg) apresenta melhores resultados. Dois concluíram que a dose padrão ou doses superiores apresentam o mesmo desfecho de sobrevida. Nenhum estudo demonstrou melhora na sobrevida com superdosagens de epinefrina.

Estudo prospectivo com 111 crianças concluiu que doses superiores a 0,015mg/kg administradas para ritmos não chocáveis podem causar fibrilação ventricular secundária, que tem desfecho pior do que a fibrilação ventricular primária.^23^

Estudo prospectivo, randomizado, duplo-cego, comparou doses elevadas de epinefrina (0,1mg/kg) com epinefrina de dose padrão como terapia de resgate para parada cardiorrespiratória hospitalar em crianças após falha de uma dose inicial padrão (n=68).^24^Os dois grupos de tratamento não diferiram significativamente em termos de taxa de retorno da circulação espontânea (que ocorreu em 20 pacientes no grupo de doses elevadas, e em 21 do grupo de dose padrão).^24^Contudo, a taxa de sobrevivência em 24 horas foi menor no grupo de dose elevada de epinefrina como terapia de resgate quando comparada ao grupo de dose padrão.^24^Um dos 34 pacientes do grupo com altas doses sobreviveu durante 24 horas, em comparação com 7 dos 34 pacientes no grupo de dose padrão (p=0,05).^24^

Estudo retrospectivo comparou dose padrão, doses baixas e doses altas de epinefrina na parada cardiorrespiratória pediátrica. Não houve sobrevida imediata ou após 24 horas no grupo que recebeu dose inferior a 0,0018mg/kg.^25^No grupo que recebeu doses acima da padrão, não houve sobrevida imediata em crianças que receberam mais de 0,0357mg/kg, e nem houve sobrevida em 24 horas em crianças que receberam dose superior a 0,019mg/kg.^25^

Estudo controlado randomizado multicêntrico conduzido em sete hospitais com 230 pacientes menores de 22 anos comparou dose padrão (n=86) com dose dez vezes maior de epinefrina (n=127).^26^Não houve diferença estatística significativa entre os dois grupos quantos ao desfecho (retorno da circulação espontânea, sobrevida em 24 horas, sobrevida à alta e prognóstico neurológico).^26^

Estudo prospectivo multicêntrico separou 92 crianças entre 7 dias de vida e 17 anos vítimas de parada cardiorrespiratória em dois grupos.^27^O primeiro grupo (n=12) recebeu doses padrão de epinefrina enquanto o segundo (n=80) recebia a primeira dose padrão e as demais dez vezes maior.^27^Não houve diferença nos dois grupos em relação ao retorno da circulação espontânea, ao tempo de ressuscitação total, ao estado neurológico no final do episódio e à sobrevivência à alta hospitalar e em 1 ano de acompanhamento.^27^Os estudos avaliados nesta revisão mostram que não há vantagens em modificar a dose padrão preconizada pela AHA.

## CONCLUSÃO

Há poucos artigos publicados que avaliam o uso da epinefrina na pediatria. Após avaliar os estudos desta revisão, concluímos que a epinefrina deve ser administrada na parada cardiorrespiratória em crianças o mais rápido possível. A dose deve ser a padrão (0,01mg/kg). Apesar dos estudos demonstrarem menor sobrevida com o aumento das doses de epinefrina, esse fato pode ser explicado por outros motivos, como o tempo de ressuscitação cardiopulmonar e o ritmo inicial de parada cardiorrespiratória. Apenas um estudo avaliou o intervalo entre as doses de epinefrina e concluiu que o intervalo recomendado atualmente (3 a 5 minutos) apresenta menor sobrevida quando comparado a intervalos maiores, assim como já demonstrado em adultos, sugerindo que menos epinefrina (dose cumulativa ou frequência) pode ser benéfico na parada cardiorrespiratória pediátrica intra-hospitalar.

Mais estudos precisam ser realizados para um melhor entendimento do ajuste de intervalo e o número máximo de doses de epinefrina − preferencialmente ensaios clínicos aleatorizados.
